# Phosphorylation and function of DGAT1 in skeletal muscle cells

**DOI:** 10.1007/s41048-015-0004-1

**Published:** 2015-08-21

**Authors:** Jinhai Yu, Yiran Li, Fei Zou, Shimeng Xu, Pingsheng Liu

**Affiliations:** National Laboratory of Biomacromolecules, Institute of Biophysics, Chinese Academy of Sciences, Beijing, 100101 China; University of Chinese Academy of Sciences, Beijing, 100049 China; Department of Biological Science and Biotechnology, School of Biological Science and Medical Engineering, Beihang University, Beijing, 100191 China; Graduate School of Anhui Medical University, Hefei, 230032 China; General Hospital of Air Force, Beijing, 100142 China

**Keywords:** DGAT1, Phosphorylation, Single point mutation, Enzymatic activity

## Abstract

Aberrant intramuscular triacylglycerol (TAG) storage in human skeletal muscle is closely related to insulin insensitivity. Excessive lipid storage can induce insulin resistance of skeletal muscle, and under severe conditions, lead to type 2 diabetes. The balance of interconversion between diacylglycerol and TAG greatly influences lipid storage and utilization. Diacylglycerol O-acyltransferase 1 (DGAT1) plays a key role in this process, but its activation and phosphorylation requires further dissection. In this study, 12 putative conserved phosphorylation sites of DGAT1 were identified by examining amino acid conservation of DGAT1 in different species. Another 12 putative phosphorylation sites were also found based on bioinformatics predictions and previous reports. Meanwhile, several phosphorylation sites of DGAT1 were verified by phosphorylation mass spectrometry analysis of purified DGAT1 from mouse myoblast C2C12 cells. Using single point mutations, a regulatory role of 3 putative phosphorylation sites was dissected. Finally, using truncation mutations, a potential domain of DGAT1 that was involved in the regulation of enzymatic activity was revealed. This study provides useful information for further understanding DGAT1 activity regulation.

## Introduction

Triacylglycerol (TAG) is the main energy storage compound in mammals. Due to their hydrophobicity, TAG has an energy density six times greater than hydrated glycogen (Yen et al. 2008). TAG is the main component of plant seed oils, which are important for the human diet as well as in industrial applications. Furthermore, TAG from plants and microorganisms can be transformed to biofuels.

In mammals, TAG is mainly stored in adipocytes but also stored in mammary epithelial cells, hepatocytes, enterocytes, and myocytes. TAG synthesized and stored in hepatocytes and enterocytes are used primarily for lipoprotein assembly and secretion. In myocytes, TAG serves as energy storage, where they are hydrolyzed to free fatty acids (FFAs) to provide substrates for mitochondrial β-oxidation and the production of adenosine triphosphate (ATP).

However, ectopic storage of TAG is closely related to human diseases, such as obesity, diabetes, and steatohepatitis. Insulin-regulated glucose metabolism is mainly accomplished by skeletal muscle in humans (Shulman et al. 1990), and insulin resistance (IR) in skeletal muscle is a main cause of type 2 diabetes (T2D). There is substantial evidence, suggesting that ectopic storage of TAG in myocytes is intimately related to IR (Kelley and Goodpaster 2001; Kelley et al. 2002; Machann et al. 2004; Moro et al. 2008; Perseghin et al. 1999). For example, knock out of acyl-CoA:diacylglycerol acyltransferase 1 (DGAT1) in mice effectively inhibited TAG accumulation induced by high fat diet and also alleviated IR (Chen et al. 2002). In another study, DGAT1 knockout increased insulin sensitivity of skeletal muscle and white fat tissue in mice fed a standard diet (Chen et al. 2004). However, it has been observed that the skeletal muscle of endurance athletes contains high levels of TAG storage but is acutely sensitive to insulin (Goodpaster et al. 2001; Russell et al. 2003). Similarly, another study found that acute exercise increased TAG synthesis while suppressing IR (Schenk and Horowitz 2007).

Although the precise mechanisms linking TAG storage in skeletal muscle and insulin sensitivity are not known, enzymes that synthesize TAG from diacylglycerol (DAG) and acetyl-CoA charged fatty acids are most likely involved in this process. *Dgat1* and *Dgat2* are the two main genes encoding the enzymes for this process (Cases et al. 1998, 2001). Although they catalyze the same reaction, the two enzymes belong to different families, have little sequence similarity, and have distinct tissue distributions. DGAT1 plays a major role in TAG synthesis skeletal muscle (unpublished data).

In most species, *Dgat1* encodes a protein of ~500 amino acids with molecular weight ~55 kDa. DGAT1 contains a large hydrophobic domain, localizes in the endoplasmic reticulum (ER), can form homo-tetramers through N-terminal interactions (Yen et al. 2008), and has a potential DAG binding site close to the C terminus. DGAT1 is regulated at both transcriptional and post-transcriptional levels. The protein is predicated to contain several sites which could be phosphorylated by protein kinase A (PKA) and protein kinase C (PKC) (Yen et al. 2008). The activity of DGAT1 is regulated by phosphorylation in rat livers and adipocytes (Haagsman et al. 1981, 1982). However, human DGAT1 with a Y316H mutation overexpressed in adipocytes did not show any apparent upregulation in enzymatic activity in an in vitro assay (Yu et al. 2002). DGAT1 was shown to be regulated post-transcriptionally by observing 3T3-L1 cells; during differentiation, mRNA level of DGAT1 was not proportional to its enzymatic activity (Cases et al. 1998; Yu et al. 2002). In this study, we investigated more potential phosphorylation sites of DGAT1. We assayed the enzymatic activities of proteins carrying different mutations at these sites, providing more clues for screening drugs regulating the activity of DGAT1. We also investigated the functional domains of DGAT1 by truncating it into different fragments to delineate the mechanisms regulating DGAT1 activity.

## Results and discussion

### DGAT1 potential phosphorylation sites predication by informatics

**Fig. 1:**
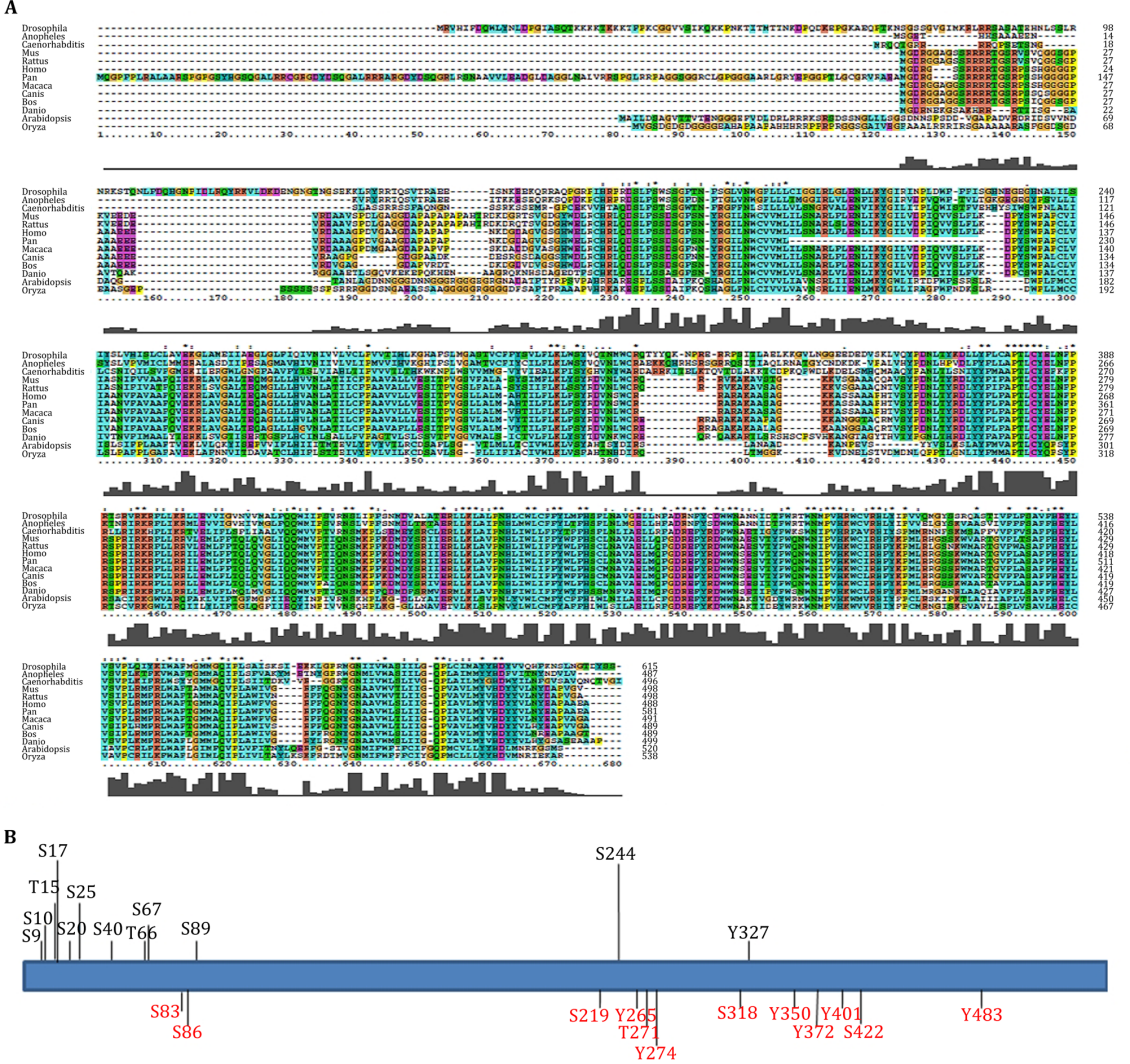
The characteristics of DGAT1 amino acid sequence. **A** DGAT1 amino acid sequence was conserved from *C. elegans* to Human. DGAT1 from selected species were from NCBI and homology alignment was analyzed by ClustalX2. **B** Putative phosphorylation sites of DGAT1 (serine, threonine, and tyrosine). *Red font* indicated sites were conserved sites; *black font* indicated sites were potential sites predicted by bioinformatics or preliminarily identified in previous studies

We compared DGAT1 amino acid sequences from 13 different species and found that DGAT1 was highly conserved from lower animals (e.g., *C. elegans*) to higher animals (e.g., human) (Fig. 1A). There were 12 potential phosphorylation sites (serine, threonine, and tyrosine) conserved across all species analyzed (Fig. 1B, red fonts indicated sites): S83, S86, S219, Y265, T271, Y274, S318, Y350, Y372, Y401, S422, and Y483 (sites in mouse DGAT1). There were another 12 potential phosphorylation sites (Fig. 1B, black fonts indicated sites) predicated by bioinformatics strategies (NetPhosK and PhosphoSitePlus) and previous literature: S9, S10, T15, S17, S20, S25, S40, S66, S67, S89, S244, and Y327 (Humphrey et al. 2013; Yu et al. 2002). Among these potential phosphorylation sites, only Y327 (human Y316) was confirmed as a phosphorylation site by mutagenesis (Yu et al. 2002), and T15 (Han et al. 2010), S17 (Huttlin et al. 2010; Kettenbach et al. 2011; Wilson-Grady et al, 2013), S20 (Han et al. 2010; Huttlin et al. 2010; Kettenbach et al. 2011; Villen et al. 2007; Wilson-Grady et al. 2013), S40 (Trinidad et al. 2012), and S67 (Huttlin et al. 2010; Wilson-Grady et al. 2013) were previously confirmed by phosphorylation mass spectrometry.

### DGAT1 protein enrichment and phosphorylation mass spectroscopy

**Fig. 2:**
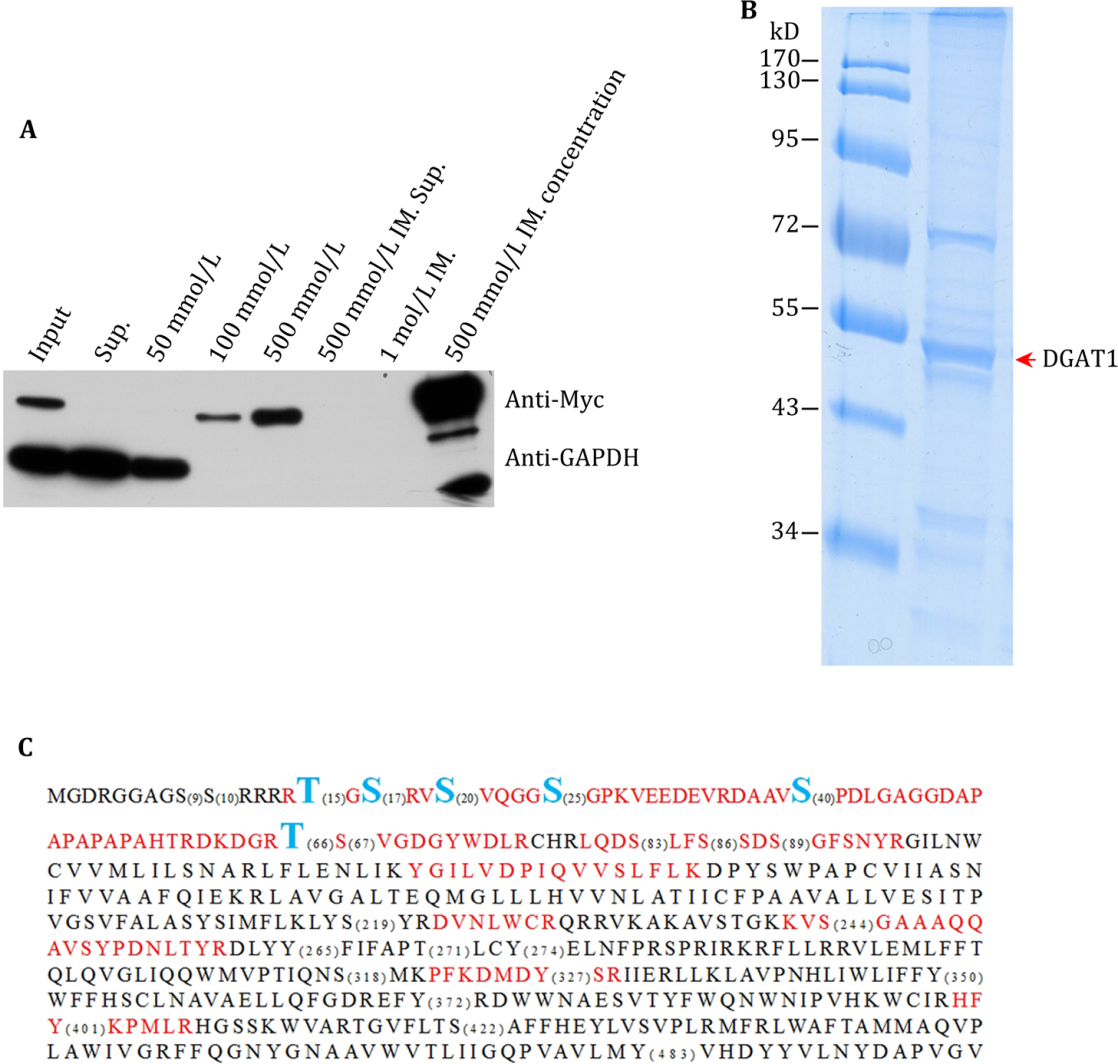
Identification of DGAT1 phosphorylation sites by phosphorylation mass spectrometry. **A** DGAT1-His was overexpressed in C2C12 cells and purified by Ni-affinity chromatography. 500 mmol/L imidazole (IM) eluted sample was concentrated for further analysis. *Sup*: supernatant. **B** Enriched DGAT1 was analyzed by SDS-PAGE and Colloidal blue staining; *red arrow* indicated enriched DGAT1-His. **C** Phosphorylation mass spectrometry of enriched DGAT1,* blue fonts* indicated detected phosphorylation sites, *red fonts* indicated detected amino acid fragments, and numbers in parenthesis indicated putative phosphorylation sites

To confirm the phosphorylation of potential sites predicated by bioinformatic methods and to identify novel phosphorylation sites, we analyzed the enriched DGAT1 by phosphorylation mass spectrometry. We overexpressed Myc-His tagged mouse DGAT1 in C2C12, enriched the protein by nickel-affinity chromatography, and confirmed with immunoblots. The DGAT1 was eluted with 500 mmol/L imidazole with no detectable cytosolic protein contamination. We further concentrated DGAT1 with ultra-filtration (Fig. 2A). Concentrated DGAT1 was separated by SDS-PAGE and visualized by Colloidal blue staining (Fig. 2B). The band containing DGAT1 was excised for phosphorylation mass spectrometry analysis. Peptides detected by mass spectrometry covered 1/4 to 1/3 of the sequence of DGAT1 and detected 6 phosphorylation sites: T15, S17, S20, S25, S40, and T66. The T66 site is a novel discovery, and the other sites have been reported previously, suggesting that our methods are efficient and could be used to detect further DGAT1 phosphorylation sites under different stimulation conditions.

### DGAT1 single mutagenesis and enzymatic activity assay

**Fig. 3:**
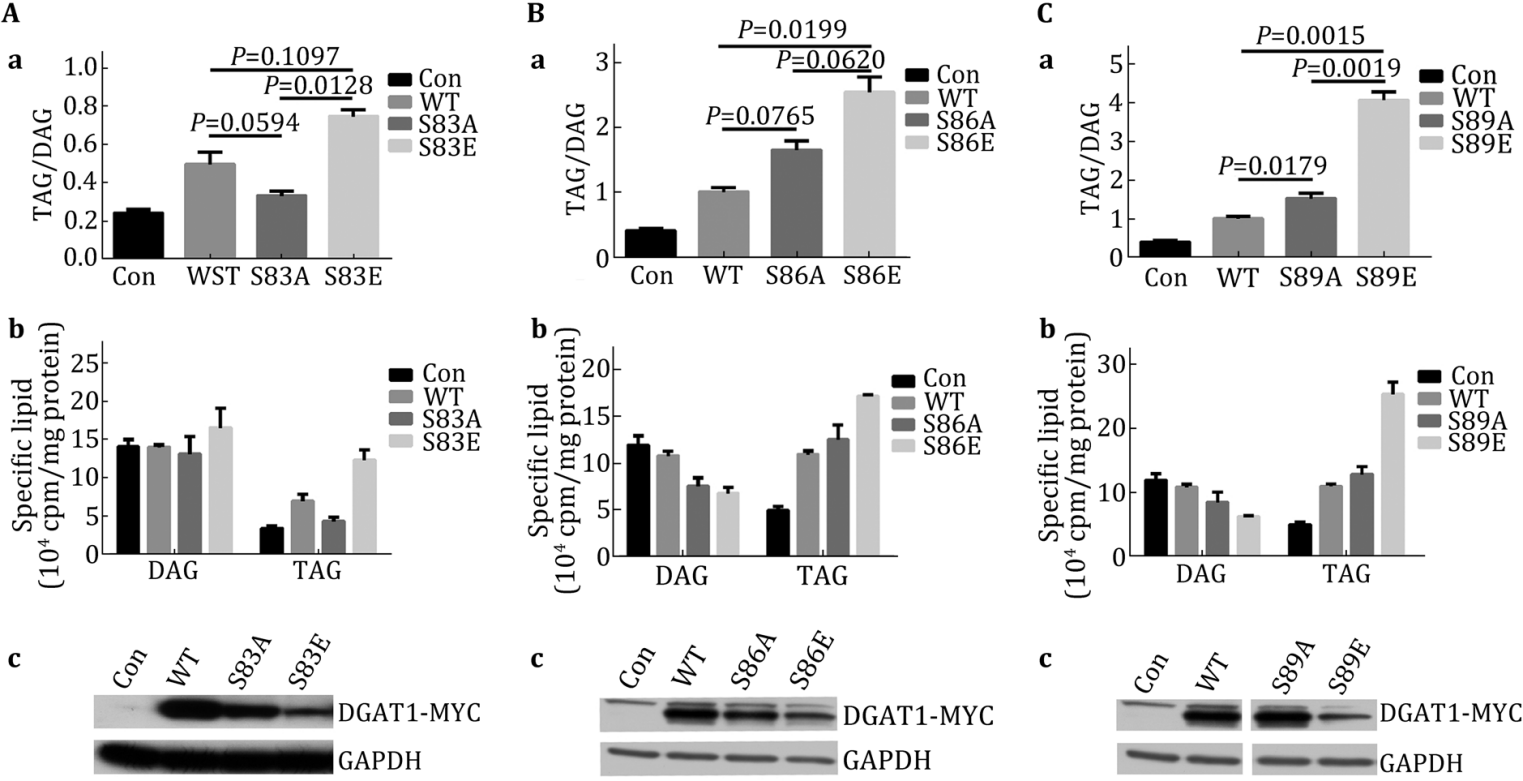
Mutagenesis of DGAT1 putative phosphorylation sites and TAG synthetic activities. **A** Mutations of DGAT1 serine 83 and their enzymatic activities, (**a**) TAG/DAG ratio of vector, DGAT1-wt, S83A, and S83E overexpressed C2C12 cells, respectively; S83A and S83E showed significant differences of enzymatic activities, (**b**) specific lipid contents of different transfections, (**c**) DGAT1-Myc/His expression level of different transfections. **B** Mutations of serine 86 and their enzymatic activities, transfection with S86E significantly increases TAG/DAG ratio compared with WT transfection. **C** Mutations of serine 89 and their enzymatic activities. S89E can significantly elevate DGAT enzymatic activity compared with WT and S89A. Data presented were analyzed by one-way ANOVA

We mutated each of the 24 potential phosphorylation sites of DGAT1 one by one to either alanine (A) or glutamate (E) to mimic dephosphorylation or phosphorylation, respectively. These mutations showed different phenotypes when overexpressed in C2C12 cells (data not shown). However, only mutations of S83, S86, and S89 could apparently regulate DGAT1 enzymatic activity (Fig. 3). Overexpression of DGAT1-S83A in C2C12 cells resulted in a lower TAG/DAG ratio compared with overexpression of wild-type DGAT1 (DGAT1-wt). Overexpression of DGAT1-S83E increased TAG/DAG ratio significantly compared with DGAT1-S83A overexpression (Fig. 3Aa). This ratio alteration suggested that the phosphorylation state of S83 might regulate DGAT1 activity.

We next compared the amount of DAG and TAG in wild type and cells overexpressing these three constructs. Although there was an increased quantity of TAG in cells overexpressing DGAT1-wt or DGAT1-S83E, the DAG content were unchanged from the control (Fig. 3Ab). This indicated that phosphorylation of S83 might also be related to the regulation of lipid uptake or DAG synthesis. One point must to be noted that the DGAT1-S83E protein was expressed much less than the other two DGAT1 proteins (Fig. 3Ac).

DGAT1-S86A overexpression did not significantly alter the TAG/DAG ratio, compared with DGAT1-wt overexpression, while DGAT1-S86E overexpression dramatically and significantly increased the TAG/DAG ratio (Fig. 3Ba). In contrast with the S83 mutations, the increase amount of TAG as a result of the overexpression of S86E mutant was accompanied by a decrease of DAG (Fig. 3Bb). Similar to the S86E, but more strikingly and significant, overexpression of DGAT1-S89E drove TAG/DAG ratio up to more than four fold (Fig. 3Bc). However, neither DGAT1-S86A nor DGAT1-S89A decreased the TAG/DAG ratio or total amount of TAGs compared to DGAT1-wt overexpression. Collectively, these data suggested that the role of these two sites in regulating enzymatic activity was much more complex and may have other regulatory input besides phosphorylation regulation.

### N-terminal of DGAT1 regulated its activity

**Fig. 4:**
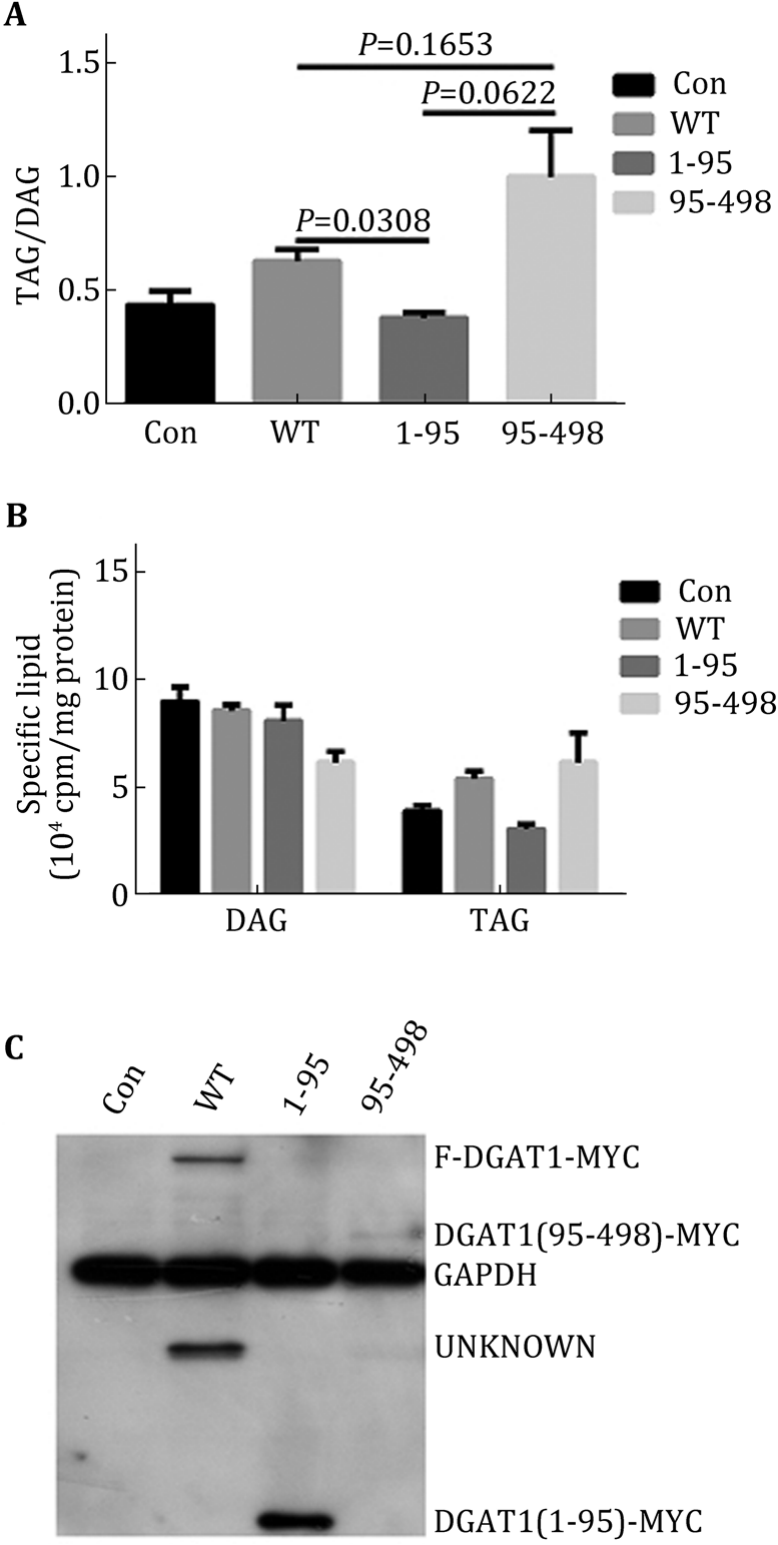
N-terminus of DGAT1 repressed its enzymatic activity. **A** TAG/DAG ratio of different transfections of DGAT1 truncation mutants in C2C12, DGAT1^95-498^ had higher enzymatic activity than WT, while DGAT1^1-95^ had no enzymatic activity compared to vector. **B** Specific lipid composition of different transfections of DGAT truncation mutants in C2C12. **C** DGAT1 expression levels of different truncation mutations detected by Myc antibody, the expression level of DGAT1^95-498^ was much lower than WT and DGAT1^1-95^

**Fig. 5:**
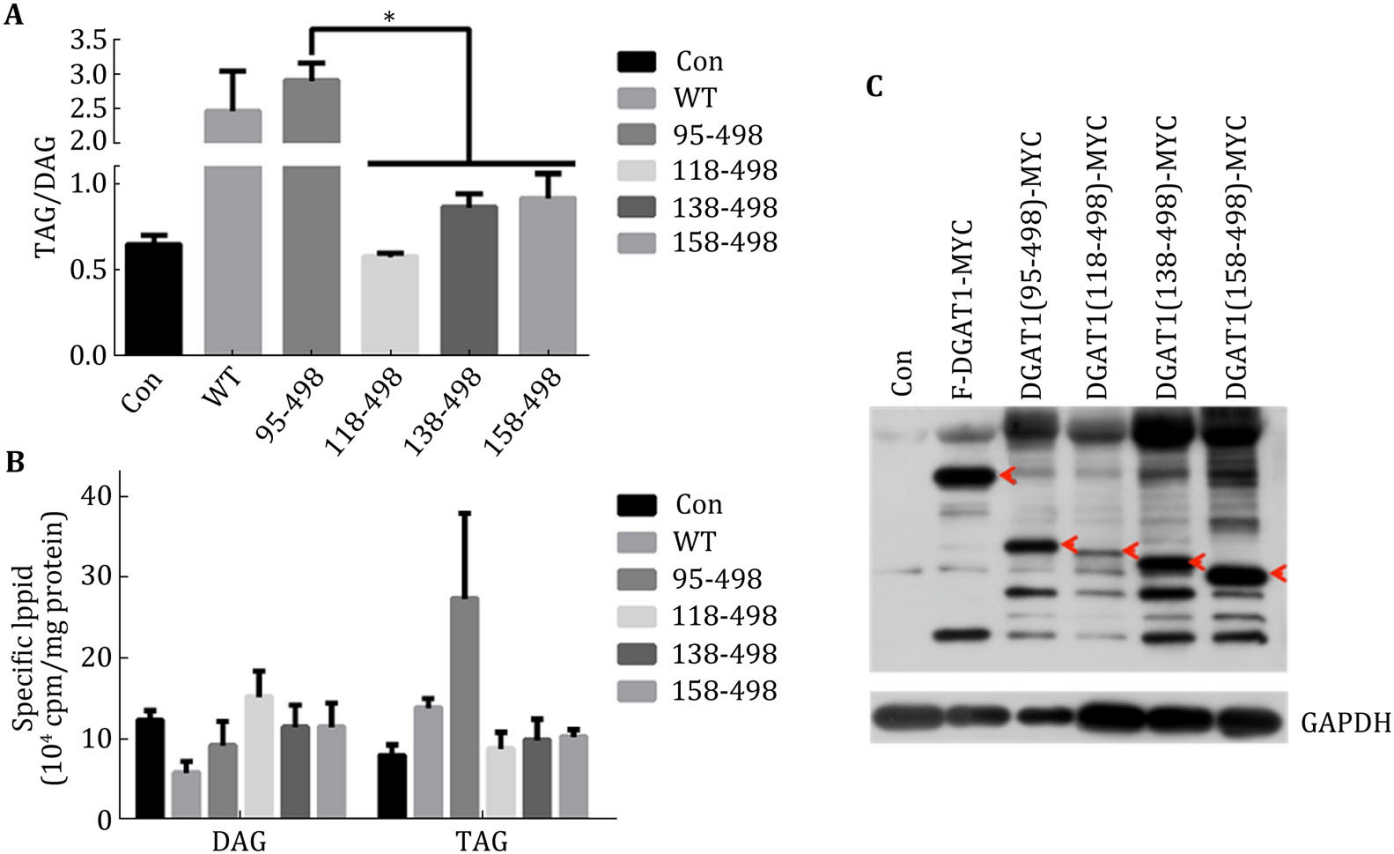
The 95-118 amino acid fragment of DGAT1 was essential to enzymatic activity. **A** TAG/DAG ratio of different transfections of DGAT1 truncations in C2C12, DGAT1^95-498^ had higher enzymatic activity than DGAT1^118-498^, DGAT1^138-498^, and DGAT1^158-498^. **B** Lipid distribution of different transfections of DGAT truncation mutants in C2C12, the TAG accumulation of DGAT1^95-498^ was greater than the other truncation mutants. **C** DGAT1 expression levels of different truncation mutants detected by Myc antibody

The point mutations of DGAT1 suggested that potential phosphorylation sites between S83 and S89 were important for regulation of DGAT enzymatic activity, and the N-terminus was assumed to have a role in oligomer formation and acyl-CoA binding (McFie et al. 2010; Siloto et al. 2008; Weselake et al. 2006). To examine the role of the N-terminus of DGAT1, we prepared two mouse DGAT1 truncation mutants including a fragment consisting of amino acids (aa) 1–95 and the other consisting of aa 95–498 according to the ClustalX2 result. Overexpression of the 1–95 fragment of DGAT1 did not alter cell TAG/DAG ratio, while overexpression of the 95–498 fragment increased the ratio of TAG/DAG compared to overexpression of DGAT1-wt (Fig. 4A). These data suggest that the N-terminus has no enzymatic activity but may negatively regulate DGAT1 activity. The total amount of TAG in cells overexpressing the 95–498 fragment was not different from cells overexpressing DGAT1-wt. However, these results may be due to the low expression level of the 95–498 fragment (Fig. 4B, C). To investigate further, we constructed another three truncation mutants randomly with about 20 aa interval: 118–498, 138–498, and 158–498. The three fragments had no apparent DGAT activity compared with DGAT1-wt and DGAT1-95–498 (Fig. 5), which suggested that aa 95–118 contained an important functional domain.

## Conclusion

The overall prevalence of diabetes and prediabetes in the Chinese adult population were estimated in 2010 to be 11.6% and 50.1%, respectively (Xu et al. 2013). Skeletal muscle accounts for the majority of glucose consumption in humans (Shulman et al. 1990), and plays a critical role in maintaining glucose homeostasis. The ectopic storage of lipids has been identified as a major cause of diabetes (Samuel and Shulman 2012), and the excess storage of lipids in skeletal muscle significantly influences its insulin sensitivity. Recently, TAG, DAG, and other bioactive lipids were proposed to be the major cause of IR in skeletal muscle (Erion and Shulman 2010). The upregulation of myocellular DGAT1 has been demonstrated to augment TAG storage, decrease cellular DAG, and protect against high fat diet induced IR (Liu et al. 2007). The activity of DGAT1 was proposed to be regulated by post-translational modification (Yu et al. 2002) and PKA might be the upstream regulator (Haagsman et al. 1981, 1982; Rodriguez et al. 1992). Several potential phosphorylation sites have been studied previously (Han 2011; Humphrey et al. 2013), but none was found to regulate DGAT1 activity by biochemical methods.

Using single point mutagenesis, we found that conserved potential phosphorylation sites (S83, S86, and S89) in the N-terminus could have a great influence on the activity of DGAT1 in C2C12 cells. When each of these three serines was mutated to glutamate to mimic phosphorylation, there was an apparent increase in DGAT1 activity, suggested that phosphorylation at each of these three sites is likely to potentiate DGAT1 activity. However, we failed to detect phosphorylation at any of these three sites by phosphorylation mass spectrometry. The biochemically enriched DGAT1 might be at basal level when lacking upstream stimulating.

Truncation of the N-terminus of DGAT1 increased enzymatic activity, suggesting that N-terminus possibly acted as a negative regulator, and the mechanisms underlying this phenomenon require further study. It is possible that the N-terminus is required to permit the formation of homo-tetramers, which may suppress enzymatic activity (McFie et al. 2010). The DGAT1 truncation mutant lacking amino acids 95–118 demonstrated that this region was important for its enzymatic activity, which suggested that this domain might contain binding sites for acyl-CoA or DAG, or other essential element to fulfill the enzymatic function. Further study of the regulatory mechanisms of DGAT1 phosphorylation would provide valuable information for screening and developing drugs to lower ectopic TAG storage in non-adipocyte tissue, with implications for diabetes, obesity, and related metabolic disorders.

## Materials and methods

### Materials

The colloidal blue staining Kit, reverse transcriptase, and pcDNA3.1(+)/myc-his A plasmid were from Invitrogen. Sodium palmitate and the TAG mixed lipid standard were from Sigma-Aldrich. Tritium labeled palmitic acid was from PerkinElmer. Myc antibody was from CST. Antibody recognized GAPDH was from Millipore. The goat anti-rabbit secondary antibody and goat anti-mouse secondary antibody were from ZSGB-BIO.

### C2C12 cell culture

Mouse C2C12 myoblasts (ATCC) were maintained in DMEM (Macgene Biotech.) supplemented with 10% FBS (Hyclone), 100 U/mL penicillin, and 100 mg/mL streptomycin (Macgene Biotech.) at 37 °C, 5% CO_2_. For plasmid transfection, C2C12 cells were trypsinized, mixed with the indicated plasmids, and nucleoporated by a standard program according to instructions from Amaxa. The transfected cells were then seeded to 12-well plates and treated cells with FFAs as described previously (Peng et al. 2011).

### Plasmid constructions

Cloning and mutagenesis primers are listed in Table 1. RNA extraction from C2C12 cells was conducted by using Trizol according to the manufacturer’s protocol (Invitrogen). All plasmids used for transfection and further experiments were proved by sequencing.

**Table 1:** Primers sequences

Primers	Forward	Reverse
pcDNA3.1-dgatl	5′-GAGAATTCGCCACCATGGGCGACCGCGGAGGCGCGGGAAGCTCTCG-3′	5′-AACTCGAGGACCCCCACTGGGGCATCGTAGTTGAG-3′
S83A	5′-CATCGTCTGCAAGATGCTTTGTTCAG-3′	5′-CATCTTGCAGACGATGGCACCTCAG-3′
SS3E	5′-ATCGTCTGCAAGATGAATTGTTCAGA-3′	5′-TTCATCTTGCAGACGATGGCACCTCAGAT-3′
S86A	5′-CAAGATTCTTTGTTCGCCTCAGACAGT-3′	5′-GCGAACAAAGAATCTTGCAGACGATG-3′
S86E	5′-AAGATTCTTTGTTCGAATCAGACAGT-3′	S′-TTCGAACAAAGAATCTTGCAGACGAT-3′
S89A	5’-TTGTTCAGCTCAGACGCTGGTTTCAGC-3’	5’-GCGTCTGAGCTGAACAAAGAATCTTG-3’
S89E	5’-TTTGTTCAGCTCAGACGAAGGTTTCAGCA-3’	5’-TTCGTCTGAGCTGAACAAAGAATCTTGCA-3’
dgatl (1-95)	5′-ATGAATTCGCCACCATGGGCGACCGCGGAGGCGC-3′	5′-ATCTCGAGACGATAATTGCTGAAACCAC-3’
dgatl (95-498)	5′-ATGAATTCGCCACCATGCGTGGTATCCTGAATTGGTG-3’	5′-ATCTCGAGGACCCCCACTGGGGCATCGT-3’
dgatl (118-498)	5′-ATGAATTCGCCACCATGATCAAGTATGGCATCCTGGT-3′	5′-ATCTCGAGGACCCCCACTGGGGCATCGT-3′
dgatl (138-498)	5’-ATGAATTCGCCACCATGTACAGCTGGCaGCCCCATG-3’	5′-ATCCGAGGACCCCCACTGGGGCATCGT-3′
dgatl (158-498)	5′-ATGAATTCGCCACCATGCAGATTGAGAAGCGCCTGGCAGTGG-3′	5′-ATCTCGAGGACCCCCACTGGGGCATCGT-3′

### FFAs treatment and lipids extraction

After C2C12 cells were transfected with the indicated plasmids and cultured for 24 h, the cells were treated with FFAs. Complete medium was pre-warmed to 60 °C and sodium palmitate was added to a final concentration of 500 µmol/L with 5 µCi/mL ^3^H-palmitic acid. The medium was cooled to 37 °C, and cells were treated for 12 h. After FFAs treatment, cells were washed three times with PBS and then lysed with 1% Triton X-100 (in PBS) at room temperature for 20 min. Cell lysates were further processed with sonication and the protein concentration determined by BCA assay (Thermo). Lipids were extracted from the cell lysates by adding 1 mL lipid extraction buffer (chloroform:methanol:acetic acid = 50:50:1, *v*/*v*/*v*). The mixture was vortexed and then centrifuged at 20,000 *g* for 10 min at 4 °C. The bottom lipid phase was transferred to fresh tubes for further analysis.

### Thin layer chromatography (TLC) and isotopic tracer assay

The extracted lipids (30 µL) were spotted onto TLC plates (Yantai Jiangyou silicone company) and developed with neutral lipids developing buffer (n-hexane:ether:acetic acid = 80:20:1, *v*/*v*/*v*). The lipids were visualized by iodine staining. The TAG and DAG fractions, identified relative to standards, were scraped into tubes. The lipids were quantified by liquid scintillation.

### DGAT1 protein enrichment and phosphorylation mass spectrometry

DGAT1-Myc-His A overexpressed C2C12 cells were harvested in lysis buffer with 5 mmol/L β-glycerophosphoric acid, 10 mmol/L NaF, 1 mmol/L Na_3_VO_4_, 10 mmol/L sodium pyrophosphate, and protease inhibitor cocktail and disrupted by sonication on ice. DGAT1 protein was enriched by Nickel-affinity chromatography (GE Health) and eluted with a step gradient of imidazole at 4 °C. The eluate was concentrated by ultra-filtration (Millipore, 30 kDa). The chromatography was followed by immunoblots. The concentrated DGAT1 proteins were separated and visualized by SDS-PAGE and Colloidal blue staining, and were excised for phosphorylation mass spectrometry analysis.

MS/MS spectra were searched by the Sequest algorithm against a database containing the mouse DGAT1 protein database and its reversed complement. Search parameters included a static modification of 57.02146 Da (carboxyamidomethylation) on Cys; dynamic modifications of 79.96633 Da (phosphorylation) on Ser, Thr, and Tyr; and 15.99491 Da (oxidation) on Met. Results were first filtered to contain only fully tryptic peptides, and then other cutoffs were established to achieve maximum sensitivity levels at 0.1% FP results using decoy matches as a guide. Sample-specific filters for the solution charge state and mass accuracy were used. A 3 Da mass tolerance window was applied separately to each analysis. After filtering by tryptic state, solution charge, and mass accuracy, only minimal filtering with Sequest scoring (XCorr and dCn) values at the level of the entire dataset was then required to achieve less than 0.1% FP rate.

### Bioinformatic analysis of DGAT1

Cross-species sequence conservation was analyzed by ClustalX2 (Larkin et al. 2007). Potential phosphorylation sites of DGAT1 were predicted by NetPhosK (Blom et al. 2004) (http://www.cbs.dtu.dk/services/NetPhosK/) and PhosphoSitePlus (Hornbeck et al. 2012) (http://www.phosphosite.org/), and the parameters were set default.

### Statistics

All experiment results were analyzed by GraphPad Prism 6.0 using one-way ANOVA.
